# Metabolic reprogramming-based characterization of circulating tumor cells in prostate cancer

**DOI:** 10.1186/s13046-018-0789-0

**Published:** 2018-06-28

**Authors:** Jing Chen, Shunwang Cao, Bo Situ, Juan Zhong, Yanwei Hu, Shufen Li, Jinlan Huang, Jiasen Xu, Shiyang Wu, Jinduan Lin, Qianwen Zhao, Zhen Cai, Lei Zheng, Qian Wang

**Affiliations:** 1 Laboratory Medicine Center, Nanfang Hospital, Southern Medical University, 1838 North of Guangzhou Avenue, Guangzhou, 510515 Guangdong China; 2 Guangdong Engineering and Technology Research Center for Rapid Diagnostic Biosensors, Nanfang Hospital, Southern Medical University, Guangzhou, Guangdong China; 3 Department of Traditional Chinese Medicine, The First People’s Hospital of Nanning, Nanning, Guangxi China; 4 Department of Clinical Laboratory, First Affiliated Hospital of Fujian Medical University, Fuzhou, Fujian China; 5 SurExam Bio-Tech, Guangzhou Technology Innovation Base, Science City, Guangzhou, Guangdong China; 6 Zhujiang Hospital, Southern Medical University, Guangzhou, Guangdong China

**Keywords:** Circulating tumor cells, Metabolic reprogramming, Cancer metastasis biomarker

## Abstract

**Background:**

Circulating tumor cells (CTCs), an advantageous target of liquid biopsy, is an important biomarker for the prognosis and monitoring of cancer. Currently, detection techniques for CTCs are mainly based on the physical and/or epithelial characteristics of tumor cells. However, biofunctional activity markers that can indicate the high metastatic capacity of CTCs are lacking.

**Methods:**

Functional microarray, quantitative real-time polymerase chain reaction, and Western blot were used on five prostate cancer cell lines with different metastatic capacities to identify the metastasis-related metabolic genes. The identified genes were detected in the CTCs of 64 clinical samples using the RNA in situ hybridization. A multi-criteria weighted model was used to determine the optimal metabolic markers for the CTCs test. Based on five fluorescent signals targeting DAPI, CD45, metabolic, epithelial (EpCAM/CKs), and mesenchymal (Vimentin/Twist) markers, the filtration-enriched CTCs were classified as GM^+^CTCs/GM^−^CTCs (metabolic types) or E-CTCs/H-CTCs/M-CTCs (EMT types). Correlation analysis and ROC curve were conducted on 54 prostate cancer samples to evaluate the clinical significance of CTCs subtypes.

**Results:**

Eight metastasis-related metabolic genes were identified, including HK2, PDP2, G6PD, PGK1, PHKA1, PYGL, PDK1, and PKM2. Among them, PGK1 and G6PD were determined as optimal glucose metabolic (GM) markers for CTCs. GM^+^CTCs (marked by PGK1/G6PD) were detectable in 64.8% (35/54) of prostate cancer patients, accounting for 46.5% (134/288) of total CTCs. An increased GM^+^CTCs level was associated with advanced tumor stage and metastasis (*P* <  0.05). In the discrimination of cancer metastasis from non-metastasis, GM^+^CTCs presented a higher AUC of the ROC curve (0.780) compared with the EMT CTCs subtypes (E-CTCs 0.729, H-CTCs 0.741, and M-CTCs 0.648). A triple tPSA–Gleason–GM^+^CTCs marker increased the AUC to 0.904, which was better than that of the tPSA–Gleason–H-CTCs marker (0.874).

**Conclusions:**

The metabolic marker (PGK1/G6PD) is determined as the indicator for the biofunctional activity analysis of CTCs, compared with the existing morphological (EMT) classification on CTCs. The metabolic characterization of CTCs demonstrates that hypermetabolic GM^+^CTCs are promising biomarkers for prostate cancer metastasis.

**Electronic supplementary material:**

The online version of this article (10.1186/s13046-018-0789-0) contains supplementary material, which is available to authorized users.

## Background

Circulating tumor cells (CTCs) are tumor cells derived from the tumor origin or metastasis and released into the bloodstream. The dissemination and tumorigenicity of CTCs [1, 2] indicate that they are important seeds for tumor metastasis, which is the leading cause of poor prognosis in cancer patients. Compared with traditional tissue biopsy, CTCs detection helps make possible non-invasive screening and monitoring of cancer. Recent studies have demonstrated the expanding roles of CTCs counts in the prognosis of breast, colorectal, lung, and prostate cancers [3–5]. However, merely detecting the number of CTCs has limited benefit for clinical interpretation when inter- and intra-tumor heterogeneities are considered [6, 7]. Further exploration of the genetic and phenotypic features of CTCs could provide information on the nature of individual tumor cells, which would be useful for disease evaluation and therapeutic decision-making.

The epithelial-mesenchymal transition (EMT) classification is the most investigated phenotypic characteristic of CTCs in recent years. EMT plays a vital role in tumor metastasis. The epithelial cells gain mesenchymal properties for cell movement via EMT, whereas the disseminated cells might recover epithelial properties for rapid colonization through mesenchymal-epithelial transition (MET) [8]. Hence, CTCs phenotypes might include E-CTCs (epithelial), M-CTCs (mesenchymal), and H-CTCs (hybrid). Increased H-CTCs and M-CTCs have been reported as more relevant to metastatic potential and aggressive progression [9, 10]. Nevertheless, static morphological analysis may be unsatisfactory for determining the change and outcome of CTCs because of the dynamic variation of EMT and MET [8]. It remains unclear whether all CTCs that present the same plastic EMT phenotype are capable of going through the metastatic cascade to form metastases. Thus, a great need exists for developing biofunctional activity markers to evaluate the actual roles of CTCs in cancer metastasis.

The deregulation of cellular energetics is a hallmark of tumor cells [11] and plays a crucial role in cancer progression by promoting EMT, anoikis resistance, angiogenesis, and cell stemness [12]. Glucose metabolic reprogramming is characterized by active aerobic glycolysis together with enhanced pentose phosphate pathway and glutaminolysis. These processes promote the accumulation of precursor molecules and activation of signaling pathways to modulate cell proliferation and migration [13, 14]. Our previous studies have suggested that tumor cell malignancy is closely associated with metabolic conversion toward a glycolytic pattern [15]. Many research works have also highlighted the decisive effect of metabolic transition on tumor cell behavior [16, 17]. For instance, the pro-metastatic protein S100A4 was reported to stimulate invasiveness in poorly motile melanoma cells by suppressing mitochondrial activity and potentiating glycolysis [16]. PCK2 is a crucial factor for the metabolic switch from oxidative phosphorylation to aerobic glycolysis. Zhao et al. [17] showed that PCK2 knockdown reduced the tumor-initiating ability of prostate cancer (PCa) cells in the in vivo xenograft models and that the increased PCK2 level was associated with more aggressive tumors and lower survival rates for PCa patients.

Accordingly, it is widely agreed that metabolic reprogramming is a crucial feature of the highly aggressive tumor cells. In the present work, we aim to utilize the metabolic characteristics to establish a functional activity indicator for CTCs, and explore the feasibility and significance of the metabolic markers in CTCs detection.

## Methods

### Cell lines and cell culture

Paired isogenic PCa cell lines PC-3 M 2B4 and PC-3 M 1E8 which differ in metastatic ability were purchased from the Institute of Basic Medical Sciences (Chinese Academy of Medical Sciences, Beijing, China). LNCAP, PC-3, and DU145 cell lines were bought from the Cell Bank of Type Culture Collection (Chinese Academy of Sciences, Shanghai, China). LNCAP and DU145 cells were maintained in Dulbecco’s Modified Eagle Medium (Gibco, Gaithersburg, MD, USA). PC-3 M 2B4, PC-3 M 1E8, and PC-3 cells were maintained in Roswell Park Memorial Institute 1640 Medium (Gibco). Cells were supplemented with 10% fetal bovine serum (Gibco) and cultured in a humidified incubator (37 °C, 5% CO_2_).

### Wound healing assay

Cell migration rates were assessed by wound healing assay. Cells were routinely cultured until they reached monolayer confluence. Wounds were created with a 200 μL tip (Corning, NY, USA). After washing with phosphate-buffered saline (Gibco), the cells were incubated with fresh serum-free medium for 24 h. The wound widths were measured using an inverted microscope (Olympus CKX41, Tokyo, Japan). The migration rate was calculated as the average wound closure percent of five random fields in three repetitions.

### Cell migration and invasion assay

Cell migration and invasion assays were performed using Transwell chambers (Corning). For the migration assays, 5 × 10^4^ cells suspended in 200 μL of serum-free medium were added to the upper chamber and 500 μL of medium containing 10% FBS was added to the lower chamber. After routine incubation for 24 h, the upper chambers were stained with a crystal violet kit (Beyotime, Shanghai, China). Cells outside the upper chamber were imaged and counted in five random fields using a microscope (Olympus Bx51, Tokyo, Japan). Similar protocols were performed for the invasion assays, except that 50 μL of Matrigel (BD Biosciences, NJ, USA) was added to the upper chamber overnight before the cells were seeded. Each assay was repeated three times.

### Microarray analysis

The gene profiles of PC-3 M 2B4 and PC-3 M 1E8 cells were compared using the Human Glucose Metabolism RT^2^ Profiler™ PCR Array (PAHS-006Z, SuperArray, Frederick MD, USA). RNA isolation and purification were conducted according to the manufacturer’s protocol (Qiagen, Hilden, Germany). Qualified RNA was converted to cDNA using the RT^2^ First Strand Kit (Invitrogen, Carlsbad, CA, USA). The cDNA template was then added to an instrument-specific ready-to-use RT^2^ SYBR Green qPCR Master Mix (Invitrogen), and the array detection was performed using an ABI PRISM7900 instrument (Applied Biosystems, Foster City, CA, USA). All Ct values were corrected by the average Ct of a housekeeping gene (ACTB). KangChen Bio-tech Company (Shanghai, China) assisted the analysis.

### Quantitative real-time polymerase chain reaction (qRT-PCR) analysis

Expression of the array-identified genes was verified in all five PCa cells by qRT-PCR. RNA was isolated using RNAiso Reagent (Takara, Dalian, China) according to the manufacturer’s instructions. RNA sample (1 μg) was used to synthesize the cDNA with a PrimeScript RT reagent kit (Takara). The qRT-PCR reaction mixture was prepared using an SYBR Premix Ex Taq kit (Takara) and detected on an ABI 7500 Fast Real-Time PCR system (Applied Biosystems). The ACTB gene was used as the internal reference. Relative quantification was calculated using the 2^−ΔΔCt^ method [18]. Gene expression results were determined as the mean of three independent tests. The information on primers is shown in Additional file 1: Table S1.

### Western blot analysis

Total proteins were extracted from the cultured cells using a Whole Cell Lysis Assay Kit (Keygen, Jiangsu, China) according to the operating instructions. Protein sample (30 μg) was separated by 15% sodium dodecyl sulphate-polyacrylamide gel (Bio-Rad, Gladesville, NSW, Australia) and transferred to polyvinylidene difluoride membranes (Millipore, Billerica, MA, USA). The membranes were then blocked with 5% non-fat milk for 2 h and incubated with primary antibodies overnight at 4 °C. After incubation with HRP-labeled secondary antibodies for 1 h at room temperature, the protein bands were detected using a chemiluminescence HRP kit (Millipore). The ACTB protein was measured as the internal reference for normalization. Experiments were repeated three times. Detailed information on the antibodies is presented in Additional file 2: Table S2.

### Patients and samples

A total of 118 cancer patients were enrolled from Nanfang Hospital, Southern Medical University (Guangzhou, China) in two cohorts from December 2016 to August 2017. Cohort 1 included 64 patients pathologically diagnosed with common cancers (12 hepatocellular carcinoma, 14 lung cancer, 10 nasopharyngeal carcinoma, 9 esophageal cancer, 6 PCa, and 13 cervical cancer). Cohort 2 included 54 patients pathologically diagnosed with PCa, of which 29 had metastatic disease and 25 had no metastatic lesions. Patients were eligible if they were over 18 years of age and had no other concurrent tumors; otherwise they were excluded. Blood samples were collected for CTCs analysis from both cohorts before any treatment was provided. Other pathological characteristics were simultaneously determined, including tumor features (Gleason score, stage, and metastasis) and disease-related serum biomarkers such as total prostate-specific antigen (tPSA), alkaline phosphatase (ALP), and Hemoglobin B (Hb).

### Ethics approval

The study methodologies conformed to the standards set by the Declaration of Helsinki. The research protocol was approved by the Ethics Committee of Nanfang Hospital (Approval No. 2016–172), and all patients provided informed consent.

### CTCs enrichment and identification

CTCs isolation and identification were performed on the CanPatrol platform (SurExam, Guangzhou, China) using size-based microfiltration and fluorescence staining, as previously described [19]. Briefly, 5 mL of blood samples were collected and pre-treated with ammonium chloride-based lysis buffer to remove erythrocytes. The karyocytes were then filtrated with a membrane (Millipore) with calibrated pores (diameter 8 μm). Cell nuclei of the retained cells were stained with 4′,6-diamidino-2-phenylindole (DAPI; Sigma, St. Louis, USA) for microscopic scanning (Zeiss, Germany). The leukocytes were excluded using the Alexa Fluor 740-labeled probes targeting CD45. Large cells with an oval or heteromorphic nucleus and specific chromatin but that had no expression of CD45 were identified as CTCs. The cell-counting results were judged as detectable CTCs (≥ 1/5 mL) and positive CTCs (≥ 3/5 mL). The method for determining the positive standard of cell counting is described in Additional file 3: Determination of the positive standard for CTCs counting.

### Detection of metabolic and EMT markers in CTCs

The multiple RNA in situ hybridization (multi-RNA-ISH) technique was used to detect the molecular characteristics of the enriched CTCs. Five single channels of the automated imaging microscope were used to detect the fluorescent signals of markers that reflect different characteristics of CTCs. Except for the above-mentioned channels for DAPI (F_1_) and CD45 (F_2_), the F_3_ channel was used to detect single or combined glucose metabolic (GM) genes. The capture probes for the previously identified genes were labeled by Alexa Fluor 647 and the probe sequences are shown in Additional file 4: Table S3. The metabolic CTCs phenotype was determined as GM^+^CTCs or GM^−^CTCs according to the selected GM markers. The Cy3-labeled epithelial markers (EpCAM and CK8/18/19) and Alexa Fluor 488-labeled mesenchymal markers (Vimentin and Twist) were detected in the F_4_ and F_5_ channel. Sequences of the capture probes for these EMT markers were described previously [19]. The EMT phenotype of CTCs was determined as epithelial- (E-), hybrid- (H-) or mesenchymal- (M-) type.

### Statistical analysis

Statistical analyses were performed using SPSS 13.0 (SPSS Inc., Chicago, IL, USA). Data were presented as mean ± SD for continuous variables (cell line assays). Differences between groups were compared using Student’s t-test or one-way ANOVA. Discontinuous variables (CTCs parameters) were presented as median with the interquartile range (IQR). Mann-Whitney U or Kruskal-Wallis H tests were used to compare differences. Categorical variables (clinical data) were expressed as numbers and percentages. The clinical relevance of the CTCs parameters was evaluated using Chi-square and Spearman’s rank correlation test. All tests were two-tailed, and a *P* value < 0.05 was considered statistically significant.

## Results

### Screening and validation of metastasis-related metabolic genes in PCa cell lines

Five PCa cell lines with different metastatic capacities were used to determine the metastatic-related glucose metabolism molecules. PC-3 M 2B4 (2B4) and PC-3 M 1E8 (1E8) are paired isogenic PCa cells with low and high metastatic abilities, both derived from PC-3 M cells [20]. We chose 2B4 and 1E8 cells for the metabolic microarray analysis. To ensure the accuracy of the following experiment, we verified the metastatic ability of these cells with wound healing and Transwell assays. The wound closure rate of 1E8 cells was (50.4% ± 8.81) at 24 h, which was much higher than that of 2B4 cells (24.1% ± 4.14). The migration and invasion numbers of 2B4 cells were (108 ± 11.2) and (78 ± 9.63), whereas the numbers for 1E8 cells were markedly higher at (345 ± 12.3) and (179 ± 11.7) (*P* <  0.001; Additional file 5: Figure S1A and B). These data verified that 1E8 cells had much higher metastatic ability than 2B4 cells, at least in vitro. LNCAP, PC-3, and DU145 cells are common PCa cells derived from PCa patients with lymphatic-, bone-, and brain-metastases, respectively [21–23]. The wound healing and Transwell assays showed that they had different metastatic capacities. The metastatic abilities of LNCAP, PC-3, and DU145 cells ranked low to high (*P* < 0.001; Additional file 5: Figure S1C and D). They were therefore used to validate the array results.

**Fig. 1:**
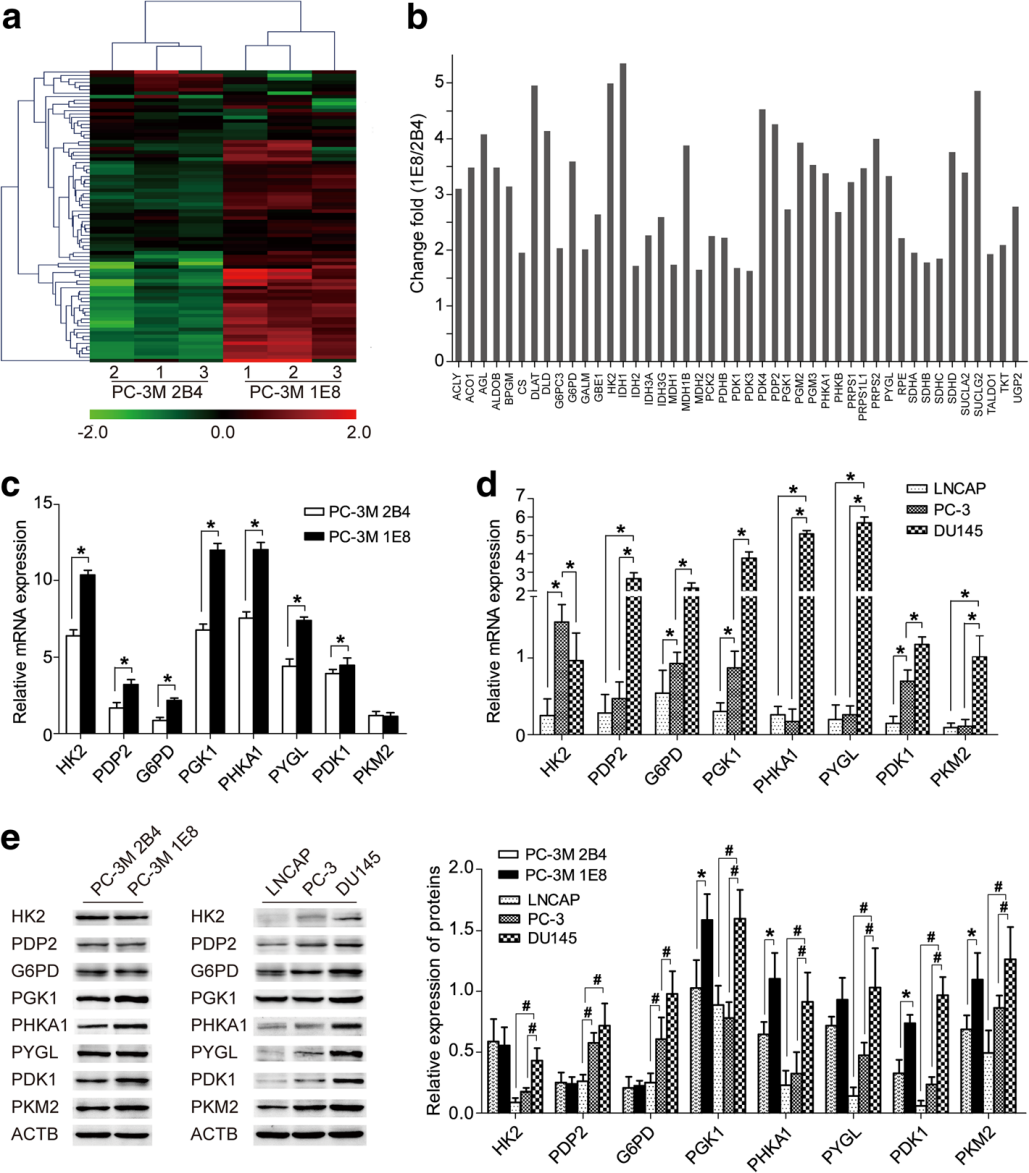
Screening and validation of metastasis-related metabolic genes in PCa cell lines. **a** Hierarchical clustering analysis of the metabolic gene profiles between 2B4 and 1E8 cells in the functional microarray assay. **b** The array identified genes that were up-regulated in 1E8 cells compared with 2B4 cells (change fold > 1.5 and *P* < 0.05). **c**–**d** Relative mRNA levels of the differentially expressed metabolic genes in **c** 2B4 and 1E8 cells, and **d** LNCAP, PC-3, and DU145 cells by qRT-PCR test, including HK2, PDP2, G6PD, PGK1, PHKA1, PYGL, PDK1, and PKM2 (^*^P < 0.05). **e** Protein levels of the metabolic genes in all five PCa cells by Western blot and quantitative analysis (^*^P < 0.05 between 1E8 and 2B4; ^#^*P* < 0.05 among LNCAP, PC-3, and DU145)

The Human Glucose Metabolism Array profiles 84 key genes involved in glucose and glycogen metabolism (Additional file 6: Table S4). We used this array to compare the metabolic genes of the low metastatic 2B4 and high metastatic 1E8 cells (Fig. 1a). Screening by change fold > 1.5 and *P* < 0.05, the array identified 45 metabolic genes that were up-regulated in 1E8 cells compared with 2B4 cells (Fig. 1b). The qRT-PCR assays verified the difference of the mRNA levels of these genes in the two cells, whereas the Western blot assays showed inconsistent results of the protein levels of some genes (Fig. 1c-e). The mRNA levels of HK2, PDP2, G6PD, and PYGL were significantly higher in the 1E8 cells than that in the 2B4 cells (*P* < 0.05), and the mRNA levels of PKM2 were similar in both cells (Fig. 1c). Nonetheless, the protein levels of HK2, PDP2, G6PD, and PYGL were similar in the two cells, and the protein level of PKM2 was significantly higher in the 1E8 cells than that in the 2B4 cells (Fig. 1e). In the comparison of LNCAP, PC-3, and DU145 cells, however, both the mRNA and protein levels of the selected genes were verified as up-regulated in the high metastatic cells (DU145) than that in the low metastatic cells (LNCAP and PC-3) (*P* < 0.05; Fig. 1d and e). Combining the literature review and these results, eight genes were further validated as showing increased mRNA expression related to the high metastatic capacity, which could be enrolled as candidates for the next assessment. These genes were HK2 (Hexokinase 2), PDP2 (Pyruvate dehydrogenase phosphatase catalytic subunit 2), G6PD (Glucose-6-phosphate dehydrogenase), PGK1 (Phosphoglycerate kinase 1), PHKA1 (Phosphorylase kinase alpha 1), PYGL (Phosphorylase-glycogen liver), PDK1 (Pyruvate dehydrogenase kinase 1), and PKM2 (Pyruvate kinase-muscle 2). Their functions in glucose metabolism are presented in Additional file 7: Figure S2. Among them, HK2, PGK1, and PKM2 participate in the transformation of glucose to pyruvate in glycolysis. G6PD catalyzes glucose-6-phosphatase to generate ribose-5-phosphate, which is the key process of the pentose phosphate pathway. PDK1 and PDP2 regulate the reaction from pyruvate to Acetyl-CoA in the tricarboxylic acid cycle. PYGL and PHKA1 are involved in glycogen degradation as the enzyme and regulator, respectively.

### PGK1 and G6PD as combined markers for the metabolic characterization of CTCs

**Fig. 2:**
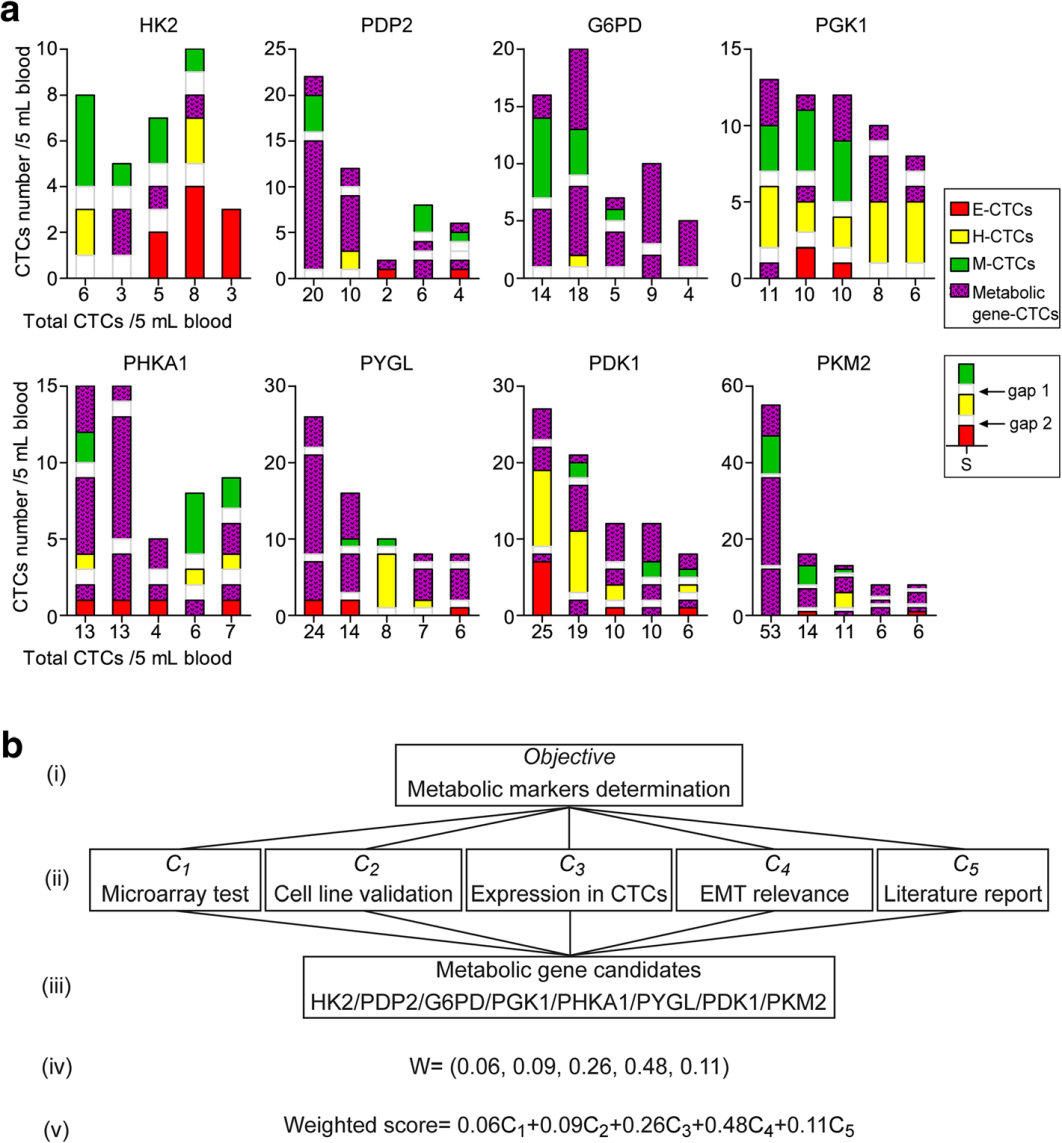
Determination of optimal markers for the metabolic profiling of CTCs. **a** The mRNA expressions of metastasis-related glucose metabolic genes in E-CTCs, H-CTCs, and M-CTCs in patients of Cohort 1, including HK2, PDP2, G6PD, PGK1, PHKA1, PYGL, PDK1, and PKM2. The legend S indicates the example that is negative for the mRNA expression of metabolic genes. Gap1 and gap2 show the intervals between different EMT subtypes; for each part, the height of gap1 or gap2 equals one CTC. **b** The analytic hierarchy process (AHP)-based model for evaluating the performance of the eight metabolic genes. (i), The target layer; (ii) the criterion layer; (iii) the scheme layer; (iv) the weighting coefficients determined by pairwise comparison matrix; and (v) the weighted calculation model of performance scores

To determine whether the changes of the metabolic genes in CTCs are in consistent with those in the cell lines, we synthesized fluorescent-labeled RNA probes for the above-identified metabolic genes to pre-test their mRNA expression in isolated CTCs. Among 64 patients diagnosed with common cancers (Cohort 1), 40 patients had positive CTCs according to the positive diagnostic criteria (≥ 3 CTCs/5 mL). The 40 positive samples were randomly and evenly divided into 8 groups for the detection of HK2, PDP2, G6PD, PGK1, PHKA1, PYGL, PDK1, and PKM2 in CTCs. We isolated and analyzed a total of 436 CTCs. The expression rates of the eight metabolic genes in CTCs were different (Table 1). Only 16% (4/25) of the CTCs expressed HK2, whereas PDP2, PYGL, and PKM2 were detected in most CTCs, presenting a percentage of 77% (30/39), 75% (44/59), and 76% (68/90), respectively. Furthermore, we assessed the mRNA expression of the metabolic genes in CTCs with different EMT subtypes. There were 77 E-CTCs, 226 H-CTCs, and 133 M-CTCs, respectively. The mRNA expression of the eight metabolic genes varied in CTCs EMT subtypes (Fig. 2a). Significant correlations were presented between the EMT phenotypes of CTCs and mRNA expression of the metabolic genes (*P* < 0.05) except PYGL (*P* = 0.141) (Table 1).

**Table 1:** The mRNA expressions of glucose metabolic markers in three EMT types of CTCs (Cohort 1^a^)

Target probe	Positive/ total CTCs	Positive/Negative in 3 types			χ^2^	*P*
		E-CTCs	H-CTCs	M-CTCs		
HK2	4/25 (0.16)	0/9	4/4	0/8	7.595	0.011^*^
PDP2	30/39 (0.77)	4/2	21/2	5/5	7.045	0.030^*^
G6PD	37/50 (0.74)	2/0	25/1	10/12	16.41	0.001^*^
PGK1	25/61 (0.41)	1/6	15/26	9/4	6.929	0.031^*^
PHKA1	28/42 (0.67)	7/4	17/2	4/8	10.98	0.004^*^
PYGL	44/59 (0.75)	5/5	27/8	12/2	3.925	0.141
PDK1	45/70 (0.64)	5/9	23/11	17/5	6.761	0.034^*^
PKM2	68/90 (0.76)	16/2	36/4	16/16	17.18	0.001^*^

^*^P < 0.05

^a^Cohort 1 includes 64 patients pathologically diagnosed with common cancer

These differences prompted us to further determine the optimal markers for CTCs metabolic profiling. The analytic hierarchy process (AHP) is a common method of multi-criteria decision analysis, in which a hierarchical system is designed to calculate the relative weights of the criteria and assess the project candidates [24, 25]. Here, we established an AHP-based weighted model for the above metabolic genes (Fig. 2b). The evaluation criteria included the expression levels revealed by microarray, cell validation results, positive expression rates in CTCs, correlations with the EMT phenotypes of CTCs and metastasis-related functions reported by literature. The weighting coefficients of these criteria were determined as 0.06, 0.09, 0.26, 0.48, and 0.11 by a pairwise comparison matrix (Additional file 8: Figure S3). The weighted performance scores of the eight candidates were calculated as 4.44–7.81 according to this model (Additional file 9: Table S5). PGK1 and G6PD, key enzymes involved in the metabolism of glycolysis and the pentose phosphate pathway, had the highest scores of 7.58 and 7.81, respectively. Therefore, we determined PGK1 and G6PD to be optimal markers to characterize the glucose metabolism of CTCs.

### Metabolic characterizations of CTCs in the peripheral blood of PCa patients

**Fig. 3:**
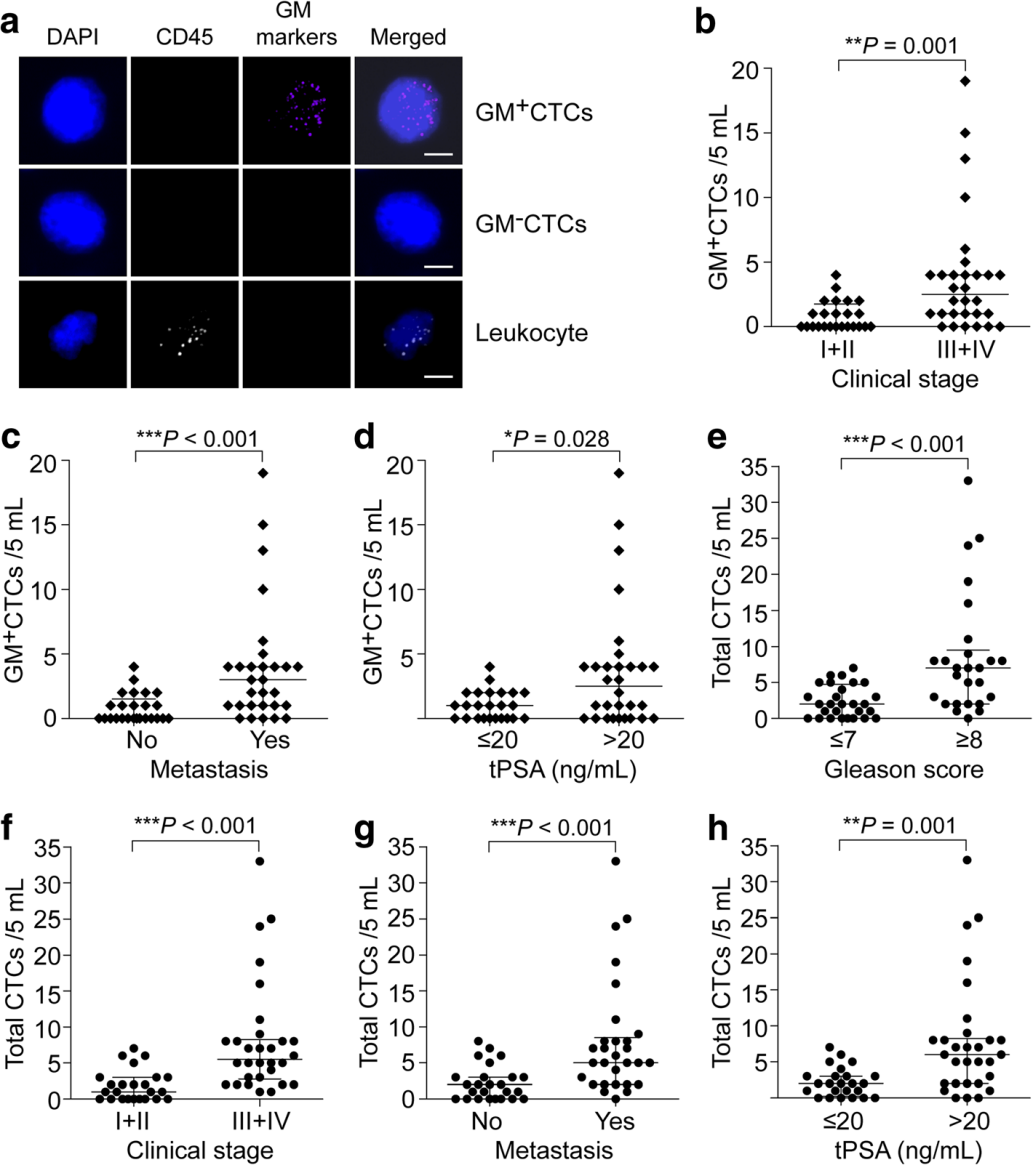
Metabolic characterizations on CTCs in the peripheral blood of PCa patients. **a** Images of multi-fluorescence signals representing the leukocyte marker (CD45) and glucose metabolic markers (GM markers: PGK1 and G6PD). The merged pattern shows the images of GM^+^CTCs, GM^−^CTCs, and leukocyte. Scale bar = 5 μm. **b**–**d** Comparison of the GM^+^CTCs numbers between the PCa patients in different subgroups of clinical characteristics, including the clinical stage (**b**), cancer metastasis (**c**), and serum tPSA level (**d**). **e**–**h** Comparison of the total CTCs numbers between the PCa patients in different subgroups of clinical characteristics, including the Gleason score (**e**), clinical stage (**f**), cancer metastasis (**g**), and serum tPSA level (**h**). ^*^P < 0.05, ^**^P < 0.01, ^***^P < 0.001

Bone metastasis is common in the newly diagnosed PCa patients even those with early stage tumors. Based on the selected PGK1 and G6PD, we collected the blood samples of 54 pathologically diagnosed PCa patients (Cohort 2) to test the metabolic features of CTCs. The patients were aged 48–86 years, among which 25 were non-metastatic and 29 were metastatic. Table 2 summarizes other clinical data, including age, Gleason score, clinical stage, and serum disease markers (tPSA, Hb, and ALP). We synthesized Alexa Fluor 647-labeled probes for PGK1 and G6PD (GM markers) to classify the isolated CTCs as GM^+^CTCs and GM^−^CTCs (Fig. 3a). Among the 54 patients, 45 patients (83.3%) presented detectable CTCs (≥ 1/5 mL), with a range of 1–33/5 mL. GM^+^CTCs were detectable in 64.8% (35/54) of patients, ranging from 1 to 19/5 mL. The patients with positive (≥ 3/5 mL) total CTCs and GM^+^CTCs accounted for 55.6% (30/54) and 31.5% (17/54) of the cohort, respectively.

**Table 2:** Clinical characteristics of PCa patients (Cohort 2)^a^ and the correlation with GM^+^CTCs and total CTCs

Clinical characteristics			GM^+^CTCs		Total CTCs	
Subgroup	Range	n (Percentage)	P/N^b^	*P*	P/N	*P*
Age (years)	48–86			0.770		> 0.999
≤ 70		27 (50.0%)	9/18		15/12	
> 70		27 (50.0%)	8/19		15/12	
Gleason score	6–9			0.099		0.013^*^
≤ 7		28 (51.9%)	6/22		11/17	
≥ 8		26 (48.1%)	11/15		19/7	
Clinical Stage	NA			0.003^*^		< 0.001^*^
I + II		24 (44.4%)	2/22		7/17	
III + IV		30 (55.6%)	15/15		23/7	
Metastasis	NA			0.002^*^		0.007^*^
No		25 (46.3%)	2/23		9/16	
Yes		29 (53.7%)	15/14		21/8	
tPSA (ng/mL)	0.01–6000.0			0.003^*^		0.017^*^
≤ 20		24 (44.4%)	2/22		9/15	
> 20		30 (55.6%)	15/15		21/9	
fPSA/tPSA	2.24–50.68			0.965		0.322
≤ 15%		32 (59.3%)	10/22		16/16	
> 15%		22 (40.7%)	7/15		14/8	
ALP (U/L)	44–718			0.200		0.808
≤ 90		26 (48.1%)	6/20		14/12	
> 90		28 (51.9%)	11/17		16/12	
Hb (g/L)	71–162			0.507		0.625
≤ 120		25 (46.3%)	9/16		13/12	
> 120		29 (53.7%)	8/21		17/12	

^*^P < 0.05

^a^Cohort 2 includes 54 patients pathologically diagnosed with prostate cancer

^b^P: positive; N: negative. The positive criterion of CTCs test is ≥3/5 mL

Moreover, we investigated the correlations between the CTCs parameters (GM^+^CTCs or total CTCs) and clinical characteristics of PCa patients (Table 2). In the early stage (I + II) group, the median GM^+^CTCs was 0 (IQR: 0, 1.75)/5 mL and 8.3% of patients had positive (≥ 3/5 mL) GM^+^CTCs; whereas in advanced stage (III + IV) group, the median and positive rates of GM^+^CTCs were higher at 2.5 (IQR: 1, 4)/5 mL and 50.0% (*P* < 0.01; Fig. 3b and Table 2). The median GM^+^CTCs number in non-metastatic and metastatic patients were 0 (IQR: 0, 1.75)/5 mL and 3 (IQR: 1, 4)/5 mL (*P* < 0.001; Fig. 3c). The positive rate of GM^+^CTCs was significantly higher in metastatic patients (51.7%) than that in non-metastatic patients (8.0%) (*P* = 0.002; Table 2). We observed similar results in the comparison of tPSA ≤20 ng/mL and tPSA > 20 ng/mL groups (*P* < 0.05; Fig. 3d and Table 2). These data revealed that both the number and positive rate of GM^+^CTCs were closely correlated with the clinical stage, metastasis and tPSA level in PCa patients. Likewise, total CTCs level was associated with the higher Gleason score (≥ 8), advanced stage, metastasis and increased tPSA level (*P* < 0.05; Fig. 3e–h and Table 2). We found no significant correlation between other clinical characteristics (age, serum ALP, and Hb level) and GM^+^CTCs or total CTCs.

### Correlation between the metabolic and EMT phenotypes of CTCs

Currently, the EMT classification is the most investigated phenotypic analysis of CTCs. The multi-RNA-ISH system could also determine the EMT phenotypes of CTCs based on the E markers (EpCAM and CK8/18/19) and M markers (Vimentin and Twist). Accordingly, we classified the CTCs of the 54 PCa patients into E-CTCs (E^+^M^−^), M-CTCs (E^−^M^+^), and H-CTCs (E^+^M^+^). The clinical relevance analysis revealed that E-CTCs were closely associated with the Gleason score, tumor stage, and tPSA level of patients, whereas H-CTCs were associated with the Gleason score, tumor stage, tPSA level, and cancer metastasis (*P* < 0.05; Additional file 10: Table S6). No significant correlation emerged between M-CTCs and these clinical characteristics of the patients.

**Fig. 4:**
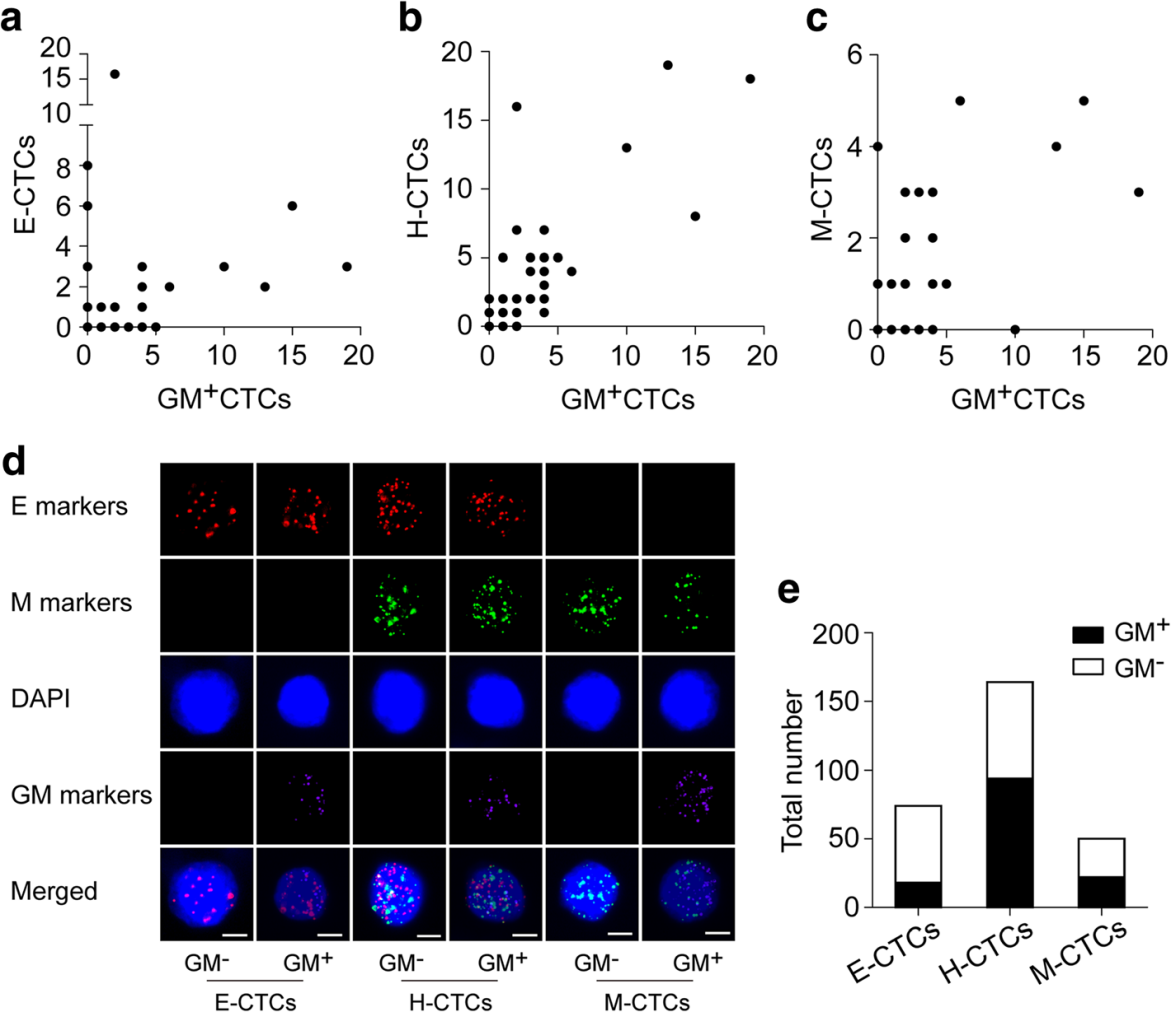
Correlation between the metabolic and EMT phenotypes of CTCs. **a**–**c** Correlation between the numbers of GM^+^CTCs and the EMT phenotypes, including E-CTCs (**a**), H-CTCs (**b**), and M-CTCs (**c**). **d** Images of multi-fluorescence signals representing the EMT markers (E markers: EpCAM and CK8/18/19; M markers: Vimentin and Twist) and glucose metabolic markers (GM markers: PGK1 and G6PD). The merged image shows the GM^+^ and GM^−^ subtypes of E-CTCs, H-CTCs, and M-CTCs, respectively. Scale bar = 5 μm. **e** The metabolic subtypes of total E-CTCs, H-CTCs, and M-CTCs

These results implied us to further evaluate the correlation between the metabolic and EMT phenotypes of CTCs. We analyzed a total of 288 CTCs in the 54 patients, among which the GM^+^CTCs accounted for 46.5% (134/288). Based on the EMT classification, the E-CTCs, H-CTCs, and M-CTCs accounted for 25.7% (74/288), 56.9% (164/288), and 17.4% (50/288) of the total CTCs, respectively. The GM^+^CTCs number was closely correlated with the number of H-CTCs (*r* = 0.807; *P* < 0.001), whereas we found medium correlations between GM^+^CTCs and E-CTCs (*r* = 0.369; *P* = 0.006) or M-CTCs (*r* = 0.553; *P* < 0.001) (Fig. 4a–c). With the multiple channels of a fluorescent microscope, we could further determine each CTC of the three EMT subtypes as GM^+^ or GM^−^ phenotype (Fig. 4d). The proportions of GM^+^CTCs in total E-CTCs, H-CTCs, and M-CTCs were 24.3% (18/74), 57.3% (94/164), and 44.0% (22/50), respectively (*P* < 0.001; Fig. 4e). These data demonstrated that the constituent ratios of GM^+^CTCs and GM^−^CTCs were different among the three EMT subtypes, indicating a remarkable correlation between the metabolic and EMT phenotypes in CTCs.

### Metabolic subtypes of CTCs indicate tumor metastasis in PCa patients

**Fig. 5:**
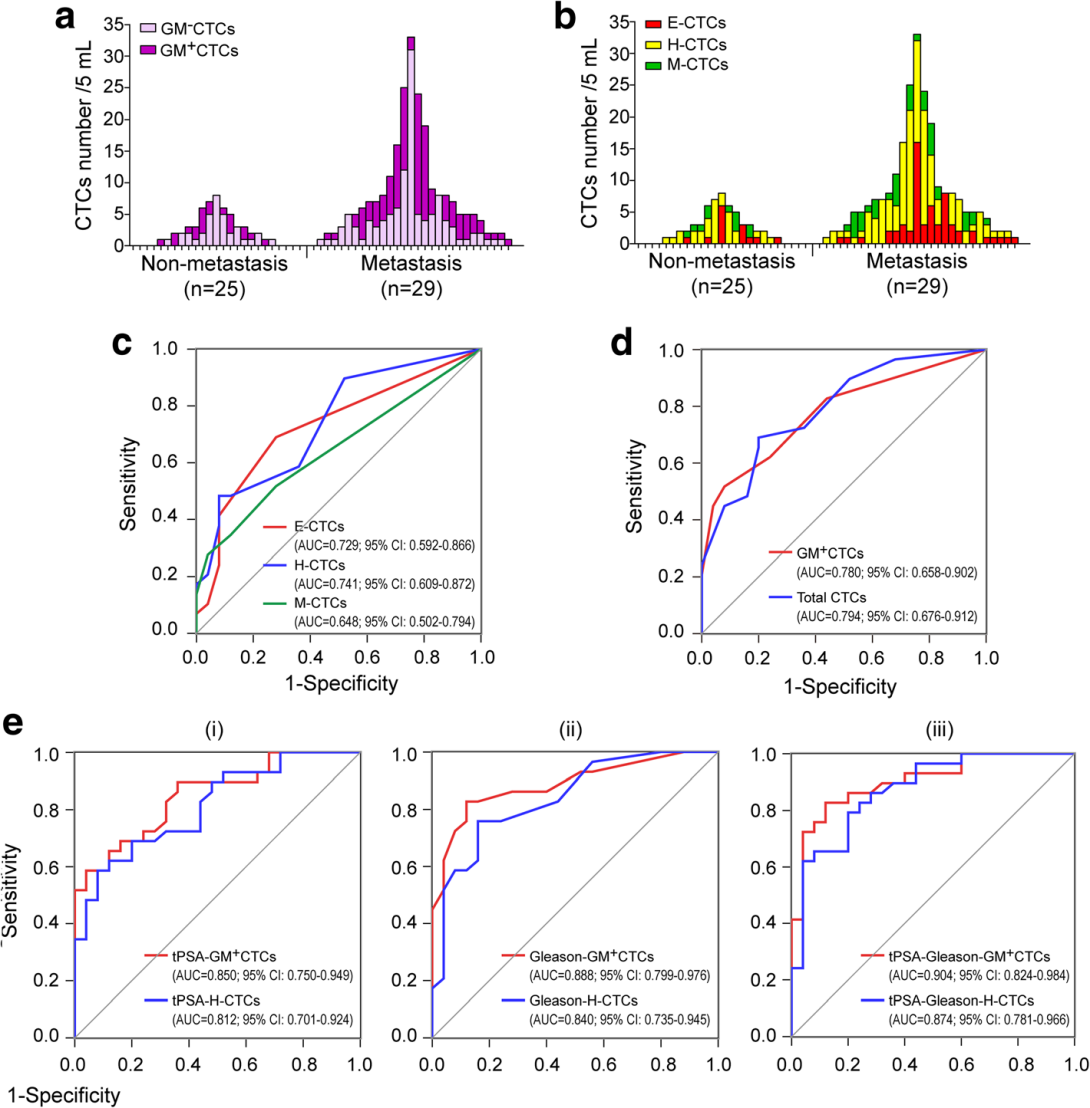
The performance of the metabolic and EMT subtype CTCs in the diagnosis of tumor metastasis. **a** Glucose metabolic classification of CTCs in non-metastatic and metastatic patients. **b** EMT phenotypes of CTCs in non-metastatic and metastatic patients. **c**–**d** Simulated ROC curves of the CTCs parameters for discriminating the metastatic PCa patients from the non-metastatic patients, including E-CTCs, H-CTCs, and M-CTCs (**c**), GM^+^CTCs and total CTCs (**d**). **e** Simulated ROC curves of the combined bigeminal (i and ii) or trigeminal (iii) markers of GM^+^CTCs and H^−^CTCs for discriminating the metastatic PCa patients from the non-metastatic patients

Some reports demonstrated that the total CTCs or mesenchymal CTCs could be indicators for cancer metastasis [26, 27]. Here, we investigated the clinical value of different CTCs parameters in the diagnosis of metastasis. In the 25 non-metastatic and 29 metastatic PCa patients, the characteristics of metabolic and EMT phenotypes on CTCs varied among individual patients (Fig. 5a-b and Additional file 11: Table S7). The area under the curves (AUC) of the receiver operating characteristic (ROC) is usually used to evaluate the efficacy of a diagnostic test by taking sensitivity and specificity into account. We therefore simulated the ROC curve to assess the performance of CTCs parameters in discriminating the metastatic PCa patients from the non-metastatic patients. The AUCs of E-CTCs, H-CTCs, and M-CTCs were 0.729, 0.741, and 0.648, respectively (Fig. 5c). The AUC of GM^+^CTCs was 0.780, which was better than that of the E-, H-, and M-CTCs but similar to that of the total CTCs (0.794) (Fig. 5d). When combining other commonly used clinical indexes, we found that the union of tPSA or Gleason score with the CTCs subtypes (GM^+^CTC or H-CTCs) improved the discrimination performance. The AUCs of the combined tPSA–GM^+^CTCs and Gleason–GM^+^CTCs markers were 0.850 and 0.888, which were higher than that of the combined tPSA–H-CTCs (0.812) and Gleason–H-CTCs (0.840) markers (Fig. 5e). A triple tPSA–Gleason–H-CTCs marker presented an AUC of 0.874, whereas the tPSA–Gleason–GM^+^CTCs marker increased the AUC to 0.904 (95% CI: 0.855–0.996) (Fig. 5e), with a sensitivity of 82.8% and specificity of 88.0%. These data demonstrated that the GM^+^CTCs could be potential biomarkers for the diagnosis of tumor metastasis with better performance than EMT-CTCs. A combination of tPSA > 20 ng/mL, Gleason score ≥ 8, and GM^+^CTCs ≥3/5 mL provides a high indication of PCa metastasis.

## Discussion

In the present study, we identified the specific metastasis-related metabolic genes and determined PGK1 and G6PD as combined GM markers for the metabolic profiling of CTCs. The GM markers that reflected the metabolic reprogramming could serve as the CTCs biofunctional activity indicators. We also evaluated the clinical significance of the metabolic characterization of CTCs and conducted comparison analysis between the metabolic CTCs phenotypes and the typical CTCs classification using EMT markers. Although the GM^+^CTCs had a correlation with the EMT phenotypes of CTCs, they presented higher AUCs than EMT-CTCs in the discrimination of metastatic patients, demonstrating their promise as indicators of PCa metastasis.

Mitochondrial oxidative phosphorylation is the main energy source for normal cells. Tumor cells, however, depend strongly on the enhanced glycolysis (even in the presence of sufficient oxygen), the pentose phosphate pathway, and glutaminolysis, resulting from the damaged mitochondrial function [11]. This reprogramming of the glucose metabolism plays a vital role in cancer metastasis because it allows tumor cells to escape normal growth processes. The Human Glucose Metabolism Array used in this study is a functional microarray specifically targeting the enzymes and regulators of the glucose and glycogen metabolism. It profiles 84 genes involved in the crucial processes of glycolysis, gluconeogenesis, the tricarboxylic acid cycle, the pentose phosphate pathway, and glycogen synthesis and degradation. The array identified eight metabolic genes differentially expressed among metastatic PCa cell lines: HK2, PDP2, G6PD, PGK1, PHKA1, PYGL, PDK1, and PKM2. The mRNA expressions of these metabolic genes increased significantly in the high metastatic PCa cells (1E8 and DU145) in comparison with the low metastatic cells (2B4, LNCAP, and PC-3). These genes play vital roles in the process of glucose and glycogen metabolism, and studies have demonstrated a remarkable association between their functions and the metastatic capacity of tumor cells [28–33], which was in line with our data. Previous research has found that overexpression of HK2 and PKM2 governs the glucose influx and sustain high levels of glycolysis to promote cancer cell migration and induce stem cell differentiation [28, 29]. PDP2 is the activator of pyruvate dehydrogenase (PDH), and the function of PDH in the cancer-associated fibroblasts was demonstrated essential for the migration ability of the co-cultured cancer cells [30]. PHKA1 and PYGL are regulators of glycogen metabolism which balances the glucose supply. Prakash and his colleges [31] demonstrated that the increased expression of PHKA1 was associated with younger ages of gastrointestinal stromal tumor patients. Hypoxia-induced activation of PYGL resulted in the enhancement of glycogen degradation and further contributed to the in vivo xenograft growth of U87 and MCF-7 cells by optimizing glucose utilization [32]. Kamarajugadda et al. [33] reported that the up-regulated PDKs and LDH in glycolysis could modulate the estrogen-related receptor gamma (ERRγ) to prolong the survival of mammary epithelial cells that break away from the matrix. This process strengthened the anoikis resistance to promote cell invasion and migration. These studies suggested the array-selected genes could be potential metabolic markers related to cancer metastasis.

Notably, in the verification assays, we observed inconsistent results between the protein and mRNA levels of HK2, PDP2, G6PD, PYGL, and PKM2 in the comparison of the 2B4 cells and 1E8 cells. Earlier studies reported the inconsistency between the protein and mRNA levels of gene expression in human tissue and cells [34, 35], which did not conform to the Central Dogma of biological genetics. The reasons for this phenomenon might be biological and technological. Biologically, the complicated post-transcriptional and translational regulations, such as alternative splicing [36], non-coding RNAs (miRNA and siRNA) interference [37, 38], and translational recoding [39] could change the protein level of the transcribed mRNA. Technologically, the characteristics of current methods might decrease the accuracy of mRNA or protein quantification. For instance, if an important splice variation or translational modification occurs in gene expression, the use of monoclonal antibodies directed to the wild-type could result in different protein information from mRNA expression [40, 41]. Further systematic experiments are needed to explore the concrete mechanism of the specific genes identified in the present study.

The AHP is one of the most widely used methods of multi-criteria decision analysis [24, 25]. It structures complex decision problems into a hierarchical system to conduct a rational and practical decision-making [42]. We found that the mRNA expression of the metabolic genes varied among individual CTCs of blood samples, which revealed that the changes in tumor cell lines might not be exactly the same as those in the peripheral CTCs because of the difference in sample types and detecting techniques. Thus, we used the AHP-based multi-criteria weighted model by which PGK1 and G6PD were determined as the optimal markers for CTCs metabolic analysis. They function as key enzymes in glycolysis and the pentose phosphate pathway, respectively. PGK1 catalyzes 1,3-diphosphoglyceric acid to generate 3-phosphoglyceric acid and ATP. G6PD catalyzes the dehydrogenation of glucose 6-phosphate to generate ribulose-5-phosphate and further produce ribose 5-phosphate and NADPH. Experimental and clinical studies have demonstrated the pivotal role of PGK1 and G6PD in cell malignance and cancer metastasis [43–50]. A mouse model study of metastatic gastric cancer demonstrated that the overexpression of PGK1 significantly increased the invasive and metastatic behavior of the implanted gastric tumors [43]. Apart from the PGK1-induced enhancement of energy supply, the mechanism might involve the stimulation of PGK1-activated oncogenic AKT/mTOR pathway in tumor cells [44]. Studies by Ahmad et al. [45] and Xie et al. [46] revealed that the PGK1 expression was up-regulated in the tissue of metastatic colon cancer and was closely correlated to the poor prognosis of hepatocellular carcinoma patients. Kowalik et al. [47] used a rat model to identify the most aggressive lesions at early phases of hepatic carcinogenesis and found that the increased G6PD expression induced aggressive preneoplastic hepatocytes. G6PD could activate the STAT3 pathway to induce the EMT process and further promote the migration and invasion of hepatocellular carcinoma cells [48]. The clinical analysis also revealed that the G6PD level is associated with high risk of recurrence and poor survival in patients with gastric [49] and renal [50] cancers. Therefore, increased activity of glycolysis and the pentose phosphate pathway (indicated by the up-regulation of PGK1 and G6PD) might reflect the active status of glucose metabolism in CTCs, which further reveals CTCs aggressiveness.

This theory was confirmed by the metabolic analysis of peripheral CTCs in PCa patients. The results demonstrated that the GM^+^CTCs marked by PGK1 and G6PD were closely associated with the patients’ clinical stage, cancer metastasis and serum tPSA level, although they made up only a small part of the total CTCs. A high baseline of total CTCs has proved relevant to the metastatic tendency and decreased survival of PCa [51, 52], whereas the reports on the associations between total CTCs and the clinical stage or serum tPSA are not exactly the same. For example, Resel et al. [53] demonstrated a positive correlation between the CTCs count and disease stage or tPSA level. On the contrary, research by Oscar et al. [51] and Tsumura et al. [54] suggested that the clinical stage and PSA level at diagnosis had no correlation with the baseline number of CTCs. The different detection methods used in these studies and the heterogeneity of CTCs population might explain the discrepancy. Here, we found that the positive rate and number of GM^+^CTCs were remarkably correlated with tumor stage and tPSA level. GM^+^CTCs ≥3/5 mL was indicative of the advanced tumor stage and increased tPSA concentration, which were both significant prognostic biomarkers for PCa patients. These data suggest that the GM^+^CTCs, as a highly aggressive subpopulation of the total CTCs, might be a more precise marker for tumor malignance and progression.

The EMT phenotype of CTCs is currently the most common indicator of cancer metastasis and prognosis [55, 56]. Previous reports proved the vital role of metabolic genes in EMT by in vivo and in vitro experiments. Wu et al. [57] investigated the effect of G6PD knockdown on A549, MDCK cells, and zebrafish embryos. They observed morphological changes and suppression of epithelial markers such as E-cadherin. A subcellular localization study found that the nuclear translocation of PKM2 regulated the EGF-induced EMT by promoting the transactivation of β-catenin [58]. In return, EMT can reprogram the cancer metabolic profile by activating transcription factors. Twist is a well-known transcription factor of EMT. A Twist-overexpressing assay in breast cancer cells demonstrated the up-regulation of metabolic enzymes such as LDHA, PKM2, HK2, and G6PD, which was mediated by the PI3K/AKT and p53 signaling pathways [59]. Therefore, the metabolic reprogramming and EMT, which are both crucial for the biological behavior of tumor cells, can mutually regulate each other and synergistically promote cancer metastasis. Here, our work found that the H-CTCs and M-CTCs presented higher GM^+^CTCs proportions and GM^+^CTCs correlation coefficients than those in the E-CTCs. This result also verified the significant interrelation between the metabolic and EMT phenotypes of CTCs.

Recent studies on gastric [10], colorectal [26] and breast [27] cancers demonstrated that M-CTCs rather than E-CTCs were more relevant to tumor progression and metastasis. Nonetheless, inconsistent with these reports, our results revealed that the H-CTCs but not E-CTCs or M-CTCs were significantly correlated with the metastasis of PCa. This case has two possible explanations. On the one hand, EMT is dynamic, and the reversible EMT-MET occurred during the dissemination and metastasis of tumor cells [60]. This phenomenon might result in the diversity of cell biological activities and functions even though they present the same morphological EMT phenotype. On the other hand, the importance of epithelial plasticity to metastasis has recently attracted considerable attention. A study by Ruscetti et al. [61] found that although mesenchymal and hybrid CTCs increased tumorigenesis in transgenic mouse models, only hybrid and epithelial CTCs formed macro-metastases, whereas mesenchymal CTCs persisted as micro-metastatic foci. In fact, hybrid CTCs may represent the most plastic tumor cells because they have both the epithelial and mesenchymal plasticity [62]. Therefore, the hybrid CTCs might have the greatest potential to contribute to cancer metastasis. Our next investigations showed that the GM^+^CTCs number was closely correlated with the number of H-CTCs (*r* = 0.807), whereas correlations between GM^+^CTCs and E-CTCs (*r* = 0.369) or M-CTCs (*r* = 0.553) were moderate. These data also supported the highlighted significance of hybrid CTCs in cancer metastasis. Besides, the metabolic analysis of CTCs provides a way to help settle the controversial outcomes of EMT subtypes. The aggressiveness of malignant cells might depend more on the metabolic activity as a functional indicator, compared with the morphological EMT features. We used the simulated ROC to assess the performance of EMT-CTCs and GM^+^CTCs in the discrimination of metastatic PCa patients. The AUCs of GM^+^CTCs were higher than that of H-CTCs, no matter as a single marker or as combined markers with tPSA and the Gleason score. These results demonstrate that the hypermetabolic CTCs could be a more accurate marker than EMT-CTCs for revealing the metastasis and disease progression of cancer patients.

In addition, the multi-RNA-ISH technique used in this method not only offers sensitive and visualized detection of the metabolic markers, but could also facilitate the integrated CTCs characterization with the combination of EMT phenotypes. Simultaneously profiling the metabolic and EMT subtypes would improve the molecular analysis of CTCs from the functional and morphological aspects, and further contribute to the understanding of tumor transfer mechanism and better application of CTCs tests. Nevertheless, some additional experiments are necessary to perfect this study. In the aspect of the fundamental theory, the biological functions of the PGK1/G6PD-positive CTCs and the related mechanisms remain to be illustrated using in vitro culture assays. In terms of research methodology, new techniques for the protein detection of the metabolic markers in CTCs need to be developed and tested for clinical applications. Moreover, an expanded sample size of PCa and other carcinomas would also be helpful to confirm the clinical significance of this method. Follow-up investigations on the prognostic and monitoring roles of specific metabolic CTCs phenotype will be conducted in the future.

## Conclusions

This work identifies the metabolic markers (PGK1/G6PD) as the biofunctional activity indicator for CTCs. On the basis of the existing morphological (EMT) classification of CTCs, we have established a metabolic characterization method and evaluated the clinical significance in PCa patients. The PGK1/G6PD-marked hypermetabolic CTCs (GM^+^CTCs) are promising biomarkers for the diagnosis of cancer metastasis. A combination of tPSA > 20 ng/mL, Gleason score ≥ 8, and GM^+^CTCs ≥3/5 mL provides a high indication of PCa metastasis, with a sensitivity of 82.8% and specificity of 88.0%.

## Additional files

Additional file 1: **Table S1.** Primer sequences for qRT-PCR. (DOCX 20 kb)

Additional file 2: **Table S2.** Antibodies used in Western blot analysis. (DOCX 21 kb)

Additional file 3: Determination of the positive standard for CTCs counting. (DOCX 133 kb)

Additional file 4: **Table S3.** Capture probes of the glucose metabolism genes used in the RNA-ISH. (DOCX 21 kb)

Additional file 5: **Figure S1.** Migration and invasion assays of the five PCa cell lines. (A–B) Representative images and statistical comparison between PC-3 M 2B4 and PC-3 M 1E8 cells in wound healing (A, 100×) and Transwell (B, 200×) assays. (C–D) Representative images and statistical comparison among LNCAP, PC-3, and DU145 cells by wound healing (C, 100×) and Transwell (D, 200×) assays. ^***^P < 0.001; Scale bar = 150 μm. (TIF 19013 kb)

Additional file 6: **Table S4.** Gene information on the Human Glucose Metabolism Array. (DOCX 19 kb)

Additional file 7: **Figure S2.** Functions of the differentially expressed genes in glucose and glycogen metabolism. These genes included HK2, PDP2, G6PD, PGK1, PHKA1, PYGL, PDK1, and PKM2. (TIF 1108 kb)

Additional file 8: **Figure S3.** The pairwise comparison matrix used in the AHP model. The weighting coefficients of the criteria layer were calculated on the basis of the maximum eigenvalue using the sum-product method. (TIF 481 kb)

Additional file 9: **Table S5.** Weighted scores of the metabolic gene candidates calculated by the AHP-based model. (DOCX 20 kb)

Additional file 10: **Table S6.** Correlation between the EMT phenotypes of CTCs and clinical characteristics of PCa patients (Cohort 2). (DOCX 24 kb)

Additional file 11: **Table S7.** Metabolic and EMT subtypes of CTCs in non-metastatic and metastatic PCa patients (Cohort 2). (DOCX 24 kb)

## Abbreviations

AHP: Analytic hierarchy process
ALP: Alkaline phosphatase
AUC: Area under the curve
CTCs: Circulating tumor cells
EMT: Epithelial-mesenchymal transition
G6PD: Glucose-6-phosphate dehydrogenase
Hb: Hemoglobin B
MET: Mesenchymal-epithelial transition
PCa: Prostate cancer
PGK1: Phosphoglycerate kinase 1
qRT-PCR: Quantitative real-time polymerase chain reaction
RNA-ISH: RNA in situ hybridization
ROC: Receiver operating characteristic
tPSA: Total prostate-specific antigen
