# Supplementary material for: Metabolic reprogramming-based characterization of circulating tumor cells in prostate cancer
Source: J Exp Clin Cancer Res. 2018 Jun 28;37:127. doi: 10.1186/s13046-018-0789-0 (PMC6025832; doi:10.1186/s13046-018-0789-0)
Supplement: Supplementary file 1 — Table S1. Primer sequences for qRT-PCR. (DOCX 20 kb) [file 13046_2018_789_MOESM1_ESM.docx]

**Table S1** Primer sequences for qRT-PCR

| Gene | Primer | 5’-3’ sequence |
| --- | --- | --- |
| HK2 | Forward | GAGCCACCACTCACCCTACT |
|  | Reverse | CCAGGCATTCGGCAATGTG |
| PDP2 | Forward | GGTAGACGCTTATACTCCAGGT |
|  | Reverse | CACATGGGGAACTGTTTAGGG |
| G6PD | Forward | CGAGGCCGTCACCAAGAAC |
|  | Reverse | GTAGTGGTCGATGCGGTAGA |
| PGK1 | Forward | TGGACGTTAAAGGGAAGCGG |
|  | Reverse | GCTCATAAGGACTACCGACTTGG |
| PHKA1 | Forward | GAAAGATGCTTGGGTCCGAGA |
|  | Reverse | GTCTGCATTCTTCCGATAGGC |
| PYGL | Forward | CAGCCTATGGATACGGCATTC |
|  | Reverse | CGGTGTTGGTGTGTTCTACTTT |
| PDK1 | Forward | CTGTGATACGGATCAGAAACCG |
|  | Reverse | TCCACCAAACAATAAAGAGTGCT |
| PKM2 | Forward | ATGTCGAAGCCCCATAGTGAA |
|  | Reverse | TGGGTGGTGAATCAATGTCCA |
| ACTB | Forward | CATGTACGTTGCTATCCAGGC |
|  | Reverse | CTCCTTAATGTCACGCACGAT |
