# Supplementary material for: Metabolic reprogramming-based characterization of circulating tumor cells in prostate cancer
Source: J Exp Clin Cancer Res. 2018 Jun 28;37:127. doi: 10.1186/s13046-018-0789-0 (PMC6025832; doi:10.1186/s13046-018-0789-0)
Supplement: Supplementary file 2 — Table S2. Antibodies used in Western blot analysis. (DOCX 21 kb) [file 13046_2018_789_MOESM2_ESM.docx]

**Table S2** Antibodies used in western blot analysis

| Antibodies | Specificity | Dilution | Brand (Catalog No.) |
| --- | --- | --- | --- |
| **Primer antibodies** |  |  |  |
| Anti-HK2 (C64G5) | Rabbit monoclonal | 1:1000 | CST (#2867) |
| Anti-PDP2 | Rabbit polyclonal | 1:500 | Abcam (ab99170) |
| Anti-G6PD (D5D2) | Rabbit monoclonal | 1:1000 | CST (#12263) |
| Anti-PGK1 | Rabbit polyclonal | 1:1000 | ABclonal (A1965) |
| Anti-PHKA1 (EPR12118) | Rabbit monoclonal | 1:500 | Abcam (ab176338) |
| Anti-PYGL | Rabbit polyclonal | 1:1000 | ABclonal (A6710) |
| Anti-PDK1 (D4Q4D) | Rabbit monoclonal | 1:1000 | CST (#13037) |
| Anti-PKM2 (D78A4) | Rabbit monoclonal | 1:1000 | CST (#4053) |
| Anti-ACTB (13E5) | Rabbit monoclonal | 1:5000 | CST (#4970) |
| **Secondary antibodies:** |  |  |  |
| Goat-anti-rabbit IgG/HRP | Goat polyclonal | 1:4000 | BioSS (bs0295) |
