# Supplementary material for: Metabolic reprogramming-based characterization of circulating tumor cells in prostate cancer
Source: J Exp Clin Cancer Res. 2018 Jun 28;37:127. doi: 10.1186/s13046-018-0789-0 (PMC6025832; doi:10.1186/s13046-018-0789-0)
Supplement: Supplementary file 3 — Determination of the positive standard for CTCs counting. (DOCX 133 kb) [file 13046_2018_789_MOESM3_ESM.docx]

**Determination of the positive standard for CTCs counting**

The threshold of positive CTCs counting was derived using the Youden Index [1]. This index reflects the authenticity of a diagnostic test by integrating the features of sensitivity (Se) and specificity (Sp). The Youden Index could therefore be used to determine the optimal cut-off value of a laboratory test [2]. The Youden Index is defined as (*J* = Se + Sp -1) and the cut-off value is chosen when the threshold presents a maximized *J* [3]. Here, we used the Laboratory Information System (LIS) of Nanfang Hospital to collect the CTCs counting data of cancer patients retrospectively. We searched for patients who had been pathologically diagnosed with cancer disease from January 2015 to June 2016 and had experienced the clinical CTCs tests before therapy. Next, we randomly enrolled the records of 50 non-metastatic patients and 50 metastatic patients (including 30 hepatocellular cancer, 10 nasopharyngeal carcinoma and 10 renal cancers for each group). The simulative receiver operating characteristic curve was obtained (Figure af3. A) using the SPSS 13.0 software to assess the significance of CTCs counting in the discrimination of cancer metastasis. On this curve, the sensitivity and specificity of a certain threshold (pot) were negatively correlated (Figure af3. B). Figure af3. C shows the calculated values of the Youden Index. The maximum Youden Index (0.56) highlighted the best threshold of CTCs counting at 2.5 (/5 mL), presenting the optimal sensitivity (0.82) and specificity (0.74). Because the threshold values derived by the software were the averages of two consecutively ordered test values, we changed the value to an integer (cell number) and determined the positive standard of CTCs counting as ≥ 3/5 mL for the following studies.


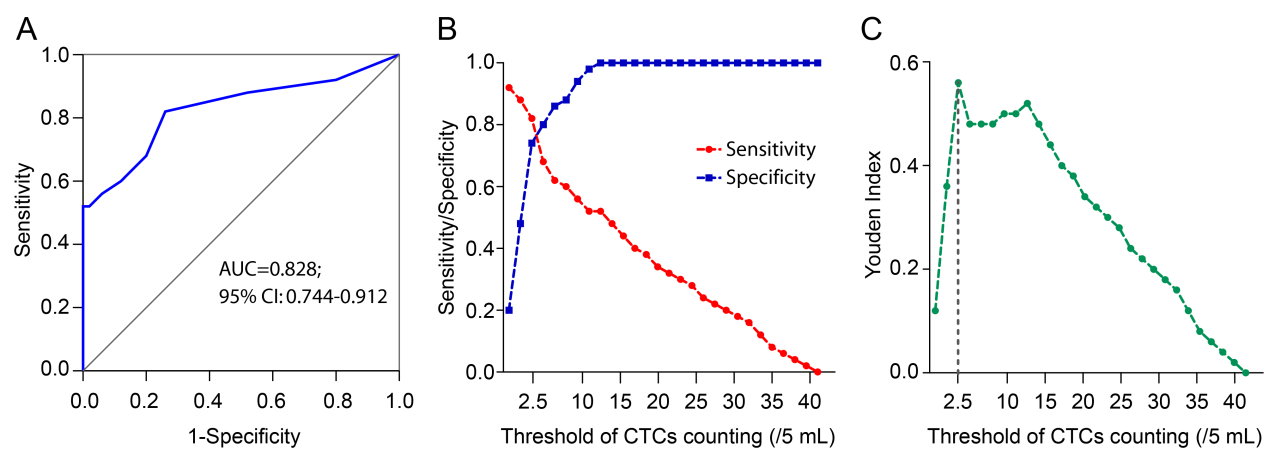


**Figure af3.** Determination of the positive standard for CTCs counting. **(A)** The simulative receiver operating characteristic curve of CTCs counting in the discrimination of cancer metastasis. **(B)** The sensitivity and specificity of the threshold values on CTCs counting. **(C)** The Youden Index (*J* = Se + Sp – 1) of the threshold values. The best threshold value was chosen when the Youden Index was maximized.

**References:**

1. Youden WJ. Index for rating diagnostic tests. Cancer. 1950;3(1):32-5.

2. Greiner M, Pfeiffer D, and Smith RD. Principals and practical application of the receiver operating characteristic analysis for diagnostic tests. Preventive Veterinary Medicine. 2000;45(1-2):23-41.

3. Grmec S and Gasparovic V. Comparison of APACHE II, MEES and Glasgow Coma Scale in patients with nontraumatic coma for prediction of mortality. Critical Care. 2001;5(1):19-23.
