# Supplementary material for: Metabolic reprogramming-based characterization of circulating tumor cells in prostate cancer
Source: J Exp Clin Cancer Res. 2018 Jun 28;37:127. doi: 10.1186/s13046-018-0789-0 (PMC6025832; doi:10.1186/s13046-018-0789-0)
Supplement: Supplementary file 4 — Table S3. Capture probes of the glucose metabolism genes used in the RNA-ISH. (DOCX 21 kb) [file 13046_2018_789_MOESM4_ESM.docx]

**Table S3** Capture probes of the glucose metabolism genes used in RNA-ISH

| Gene | Probe Sequences (5’-3’) | Gene | Probe Sequences (5’-3’) |
| --- | --- | --- | --- |
| HK2 | AAGCAGATGCGAGGCAATCA  TTGAGCTCCGTGAAGAAGTA  GCATGTGGTAGAGATACTGG  TTAGAGATCTCCAAGAGGGT  CAAGATCCAGAGCCAGGAAC  TTACTTTCACCCAAAGCACA  GGGATGGCATAGATCTGATT  GCAATGTGGTCAAACAGCTG | PHKA1 | CACCTTTCACACCAAACAGA  ACGGGGCAGTAGTGAATTTA  TACTGCAAAGGCAGGGAAGG  TGATTTCCTGTTTTGTGAGC  CATAACGACCCTGAAGCTTG  TCCATCTCGTAGAAAGCGAC  ACGATTGGGATCCTCTTTAG  GAACAATGGCCATTCACACT |
| PDP2 | TAGGACACAGTACTTGACAT  CCTTGTAGAATTTAAGATCC  CTTGCAATGTGGCAATGCTG  CATACCTGGAGTATAAGCGT  ACATGGGGAACTGTTTAGGG  CTTTGCACAGAGTAAAGCCA  GGGCTGAGTTGCAAGTGAAA  AGCTCGAAGCACTTCATTTA | PYGL | GGTTGAAACTCTTCTTCAGC  GAGGTAATATACCCTCTTGG  ATGTTCGGCCCATGTAAAAT  ATCACAGGCATTTTGCAGAC  CTATATCCAATCCAAGCTGG  TATTCATACCGAATGCCGTA  CATCTCGGATCTTCTGATTG  AATCATCTGCTTCTTCTACC |
| G6PD | GAAGTGTACGACCGTTTCCG  AAAAGCTCTTCCCGCAGGAT  CGACTGATGGAAGGCATCGC  CACCAGATGGTGGGGTAGAT  ACGATGAAGGTGTTTTCGGG  AGGAGTTGCGGGCAAAGAAG  TAGGAGGCTGCATCATCGTA  CATTCATGTGGCTGTTGAGG | PDK1 | AGCTGAAGCTGCGGCTGAAG  GAGAAGCGCGCGTAGAAGTC  CAGGAACTGCTTCATGGAGA  AACTCTTGCCGCAGAAACAT  AATAGCTTTAGCATCCTCAG  ATGTTCTTCTAGGCCTTTCA  GCATAAACTAGAGACCTGCA  TAAAGATCATCTTGCAGGCC |
| PGK1 | TTTAACGTCCAGCTTGTCCA  AGTCGACTCTCATAACGACC  TCTGGTTGTTCTTCATAGGA  TTAATCCTCTGGTTGTTTGT  AATTTGATGCTTGGGACAGC  ACTTGGCTCCATTGTCCAAG  AGGTGGCTCATAAGGACTAC  TACTTGTCAGGCATGGGCAC | PKM2 | TCACTATGGGGCTTCGACAT  CTGGGTCTGAATGAAGGCAG  ACATGTGCTCCAGGAATGTG  GTGGTGAATCAATGTCCAGG  TACAGATGATGCCAGTGTTC  TTCAACGTCTCCACTGATCG  CATTCCAGACTTAATCATCT  AGAAGTTCAGACGAGCCACA |
