# Supplementary figures and images for: Metabolic reprogramming-based characterization of circulating tumor cells in prostate cancer
Source: J Exp Clin Cancer Res. 2018 Jun 28;37:127. doi: 10.1186/s13046-018-0789-0 (PMC6025832; doi:10.1186/s13046-018-0789-0)

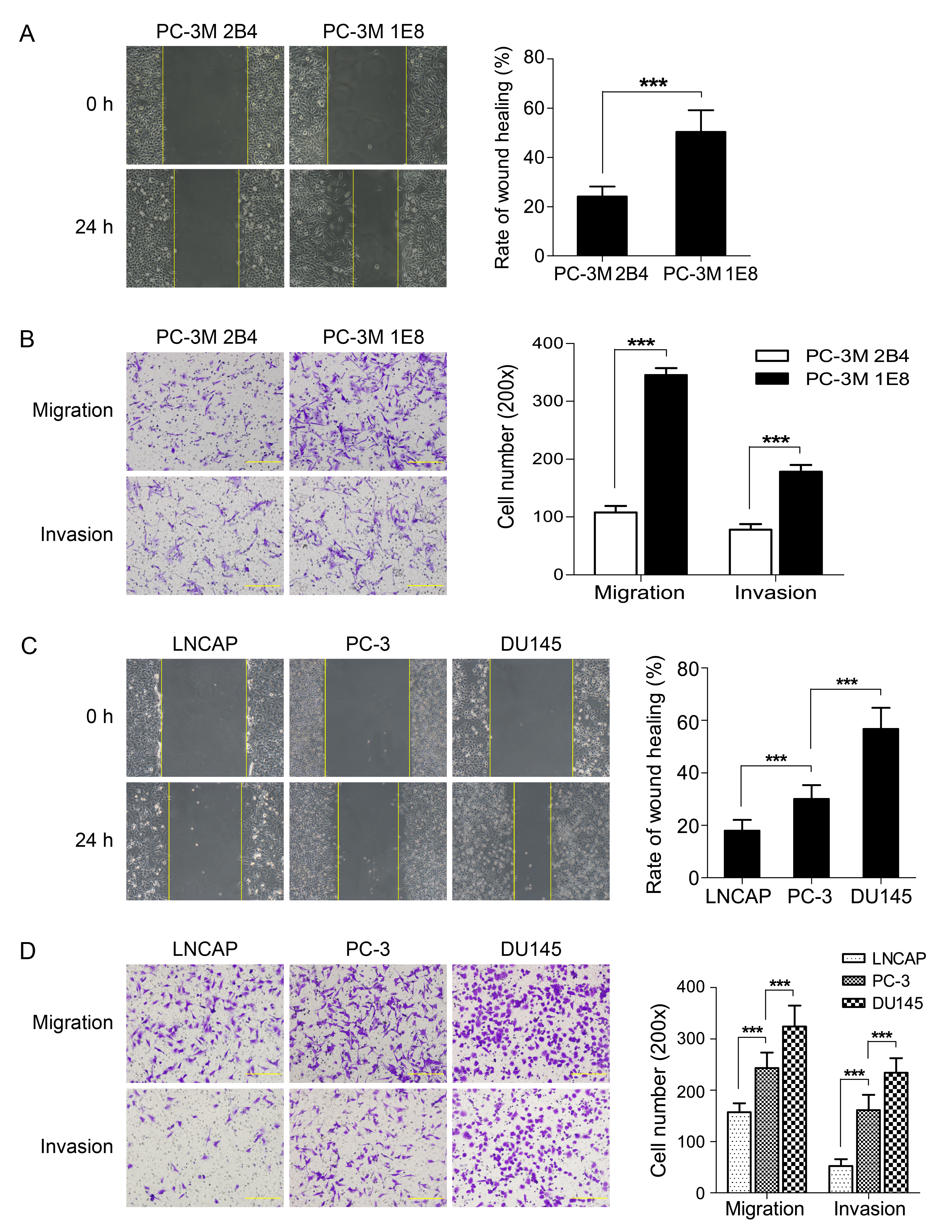

Supplement: Supplementary file 5 — Figure S1. Migration and invasion assays of the five PCa cell lines. (A–B) Representative images and statistical comparison between PC-3 M 2B4 and PC-3 M 1E8 cells in wound healing (A, 100×) and Transwell (B, 200×) assays. (C–D) Representative images and statistical comparison among LNCAP, PC-3, and DU145 cells by wound healing (C, 100×) and Transwell (D, 200×) assays. ***P < 0.001; Scale bar = 150 μm. (TIF 19013 kb) [file 13046_2018_789_MOESM5_ESM.tif]

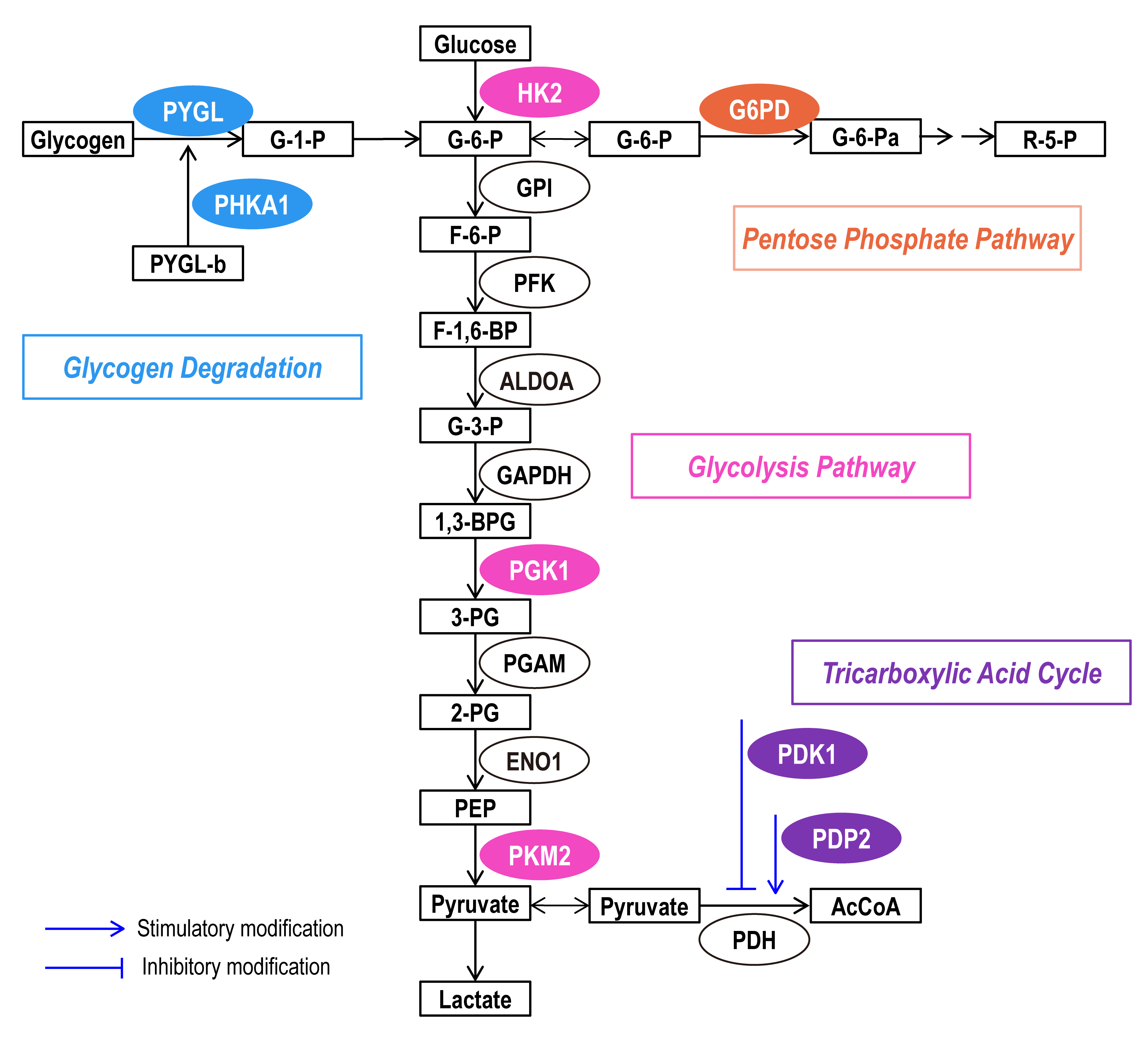

Supplement: Supplementary file 7 — Figure S2. Functions of the differentially expressed genes in glucose and glycogen metabolism. These genes included HK2, PDP2, G6PD, PGK1, PHKA1, PYGL, PDK1, and PKM2. (TIF 1108 kb) [file 13046_2018_789_MOESM7_ESM.tif]

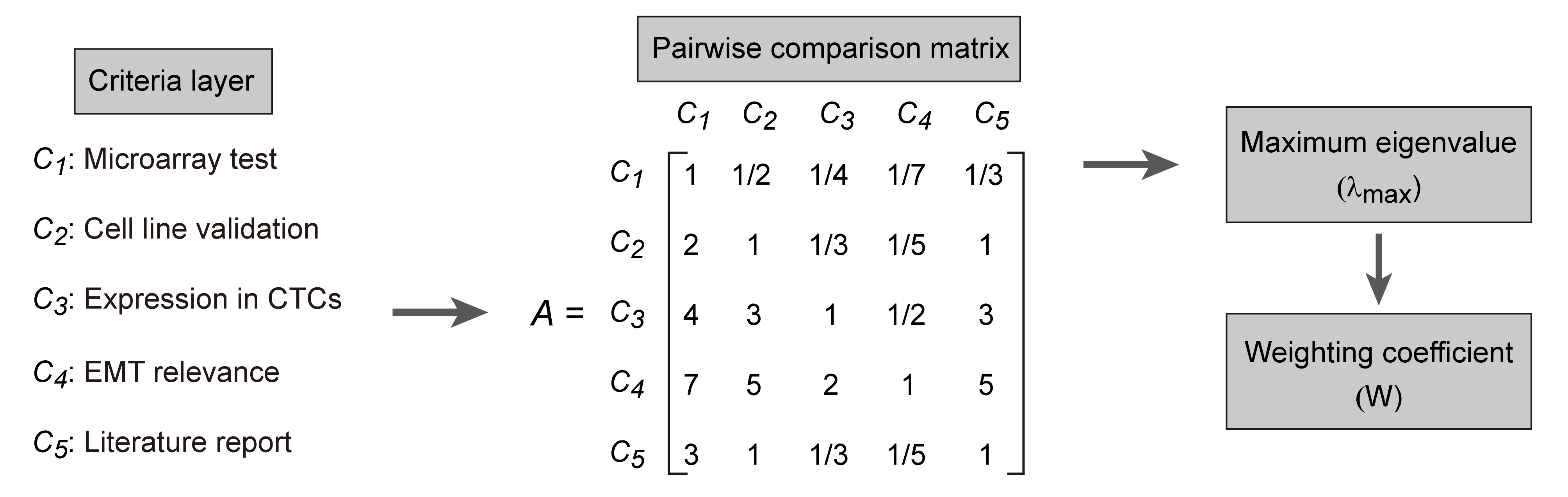

Supplement: Supplementary file 8 — Figure S3. The pairwise comparison matrix used in the AHP model. The weighting coefficients of the criteria layer were calculated on the basis of the maximum eigenvalue using the sum-product method. (TIF 481 kb) [file 13046_2018_789_MOESM8_ESM.tif]
