# Supplementary material for: Metabolic reprogramming-based characterization of circulating tumor cells in prostate cancer
Source: J Exp Clin Cancer Res. 2018 Jun 28;37:127. doi: 10.1186/s13046-018-0789-0 (PMC6025832; doi:10.1186/s13046-018-0789-0)
Supplement: Supplementary file 6 — Table S4. Gene information on the Human Glucose Metabolism Array. (DOCX 19 kb) [file 13046_2018_789_MOESM6_ESM.docx]

**Table S4** Gene information on the Human Glucose Metabolism Array

| Human Glucose Metabolism Array |
| --- |
| **Glucose Metabolism:** |
| *Glycolysis:* ALDOA, ALDOB, ALDOC, BPGM, ENO1, ENO2, ENO3, GALM, GCK, GPI, HK2, HK3, PFKL, PGAM2, PGK1, PGK2, PGM1, PGM2, PGM3, PKLR, TPI1. |
| *Gluconeogenesis:* FBP1, FBP2, G6PC, G6PC3, PC, PCK1, PCK2. |
| *Regulation:* PDK1, PDK2, PDK3, PDK4, PDP2, PDPR. |
| *TCA Cycle:* ACLY, ACO1, ACO2, CS, DLAT, DLD, DLST, FH, IDH1, IDH2, IDH3A, IDH3B, IDH3G, MDH1, MDH1B, MDH2, OGDH, PC, PCK1, PCK2, PDHA1, PDHB, SDHA, SDHB, SDHC, SDHD, SUCLA2, SUCLG1, SUCLG2. |
| *Pentose Phosphate Pathway:* G6PD, H6PD, PGLS, PRPS1, PRPS1L1, PRPS2, RBKS, RPE, RPIA, TALDO1, TKT. |
| **Glycogen Metabolism:** |
| *Synthesis:* GBE1, GYS1, GYS2, UGP2. |
| *Degradation:* AGL, PGM1, PGM2, PGM3, PYGL, PYGM. |
| *Regulation:* GSK3A, GSK3B, PHKA1, PHKB, PHKG1, PHKG2. |
