# Supplementary material for: Metabolic reprogramming-based characterization of circulating tumor cells in prostate cancer
Source: J Exp Clin Cancer Res. 2018 Jun 28;37:127. doi: 10.1186/s13046-018-0789-0 (PMC6025832; doi:10.1186/s13046-018-0789-0)
Supplement: Supplementary file 9 — Table S5. Weighted scores of the metabolic gene candidates calculated by the AHP-based model. (DOCX 20 kb) [file 13046_2018_789_MOESM9_ESM.docx]

**Table S5** Weighted scores of the metabolic gene candidates calculated by the AHP-based model

| Candidates | Scores of the criteria^a^ | | | | | Weighted scores |
| --- | --- | --- | --- | --- | --- | --- |
|  | C_1_ | C_2_ | C_3_ | C_4_ | C_5_ |  |
| W^b^ | 0.06 | 0.09 | 0.26 | 0.48 | 0.11 |  |
| HK2 | 9 | 6 | 3 | 4 | 6 | 4.44 |
| PDP2 | 8 | 8 | 8 | 4 | 3 | 5.79 |
| G6PD | 7 | 9 | 9 | 7 | 8 | 7.81 |
| PGK1 | 6 | 8 | 5 | 9 | 8 | 7.58 |
| PHKA1 | 5 | 6 | 8 | 4 | 3 | 5.17 |
| PYGL | 5 | 7 | 9 | 7 | 3 | 6.96 |
| PDK1 | 3 | 5 | 8 | 7 | 6 | 6.73 |
| PKM2 | 3 | 4 | 9 | 5 | 9 | 6.27 |

^a^The criteria of AHP: C_1_, microarray test; C_2_, cell line validation; C_3_, expression in CTCs; C_4_, EMT relevance; C_5_, literature report.

^b^W is the weighting coefficient determined by the pairwise comparison matrix.
