# Supplementary material for: Metabolic reprogramming-based characterization of circulating tumor cells in prostate cancer
Source: J Exp Clin Cancer Res. 2018 Jun 28;37:127. doi: 10.1186/s13046-018-0789-0 (PMC6025832; doi:10.1186/s13046-018-0789-0)
Supplement: Supplementary file 10 — Table S6. Correlation between the EMT phenotypes of CTCs and clinical characteristics of PCa patients (Cohort 2). (DOCX 24 kb) [file 13046_2018_789_MOESM10_ESM.docx]

**Table S6** Correlation between the EMT phenotypes of CTCs and clinical characteristics of PCa patients (Cohort 2)

| Subgroup | n | E-CTCs | | H-CTCs | | M-CTCs | |
| --- | --- | --- | --- | --- | --- | --- | --- |
|  |  | P/N^a^ | *P* | P/N | *P* | P/N | *P* |
| Age (years) |  |  | > 0.999 |  | 0.770 |  | 0.465 |
| ≤ 70 | 27 | 5/22 |  | 8/19 |  | 3/24 |  |
| > 70 | 27 | 4/23 |  | 9/18 |  | 6/21 |  |
| Gleason score |  |  | 0.001^*^ |  | 0.011^*^ |  | 0.113 |
| ≤ 7 | 28 | 0/28 |  | 4/24 |  | 2/26 |  |
| ≥ 8 | 26 | 9/17 |  | 13/13 |  | 7/19 |  |
| Clinical Stage |  |  | 0.003^*^ |  | 0.017^*^ |  | 0.066 |
| I+II | 24 | 0/24 |  | 3/21 |  | 1/23 |  |
| III+IV | 30 | 9/21 |  | 14/16 |  | 8/22 |  |
| Metastasis |  |  | 0.222 |  | 0.010^*^ |  | 0.051 |
| No | 25 | 2/23 |  | 3/22 |  | 1/24 |  |
| Yes | 29 | 7/22 |  | 14/15 |  | 8/21 |  |
| tPSA (ng/mL) |  |  | 0.003^*^ |  | 0.017^*^ |  | 0.066 |
| ≤ 20 | 24 | 0/24 |  | 3/21 |  | 1/23 |  |
| > 20 | 30 | 9/21 |  | 14/16 |  | 8/22 |  |
| fPSA/tPSA |  |  | > 0.999 |  | 0.581 |  | > 0.999 |
| ≤ 15% | 32 | 5/27 |  | 11/21 |  | 5/27 |  |
| > 15% | 22 | 4/18 |  | 6/16 |  | 4/18 |  |
| ALP (U/L) |  |  | > 0.999 |  | 0.062 |  | 0.903 |
| ≤ 90 | 26 | 4/22 |  | 5/21 |  | 5/21 |  |
| > 90 | 28 | 5/23 |  | 12/16 |  | 4/24 |  |
| Hb (g/L) |  |  |  |  | 0.939 |  | 0.625 |
| ≤ 120 | 25 | 4/21 | > 0.999 | 8/17 |  | 3/22 |  |
| > 120 | 29 | 5/24 |  | 9/20 |  | 6/23 |  |

^a^P: positive; N: negative. The positive criterion of CTCs test is ≥ 3/5 mL.

^*^*P* < 0.05.
