# Supplementary material for: Metabolic reprogramming-based characterization of circulating tumor cells in prostate cancer
Source: J Exp Clin Cancer Res. 2018 Jun 28;37:127. doi: 10.1186/s13046-018-0789-0 (PMC6025832; doi:10.1186/s13046-018-0789-0)
Supplement: Supplementary file 11 — Table S7. Metabolic and EMT subtypes of CTCs in non-metastatic and metastatic PCa patients (Cohort 2). (DOCX 24 kb) [file 13046_2018_789_MOESM11_ESM.docx]

**Table S7** Metabolic and EMT subtypes of CTCs in non-metastatic and metastatic PCa patients (Cohort 2)

| Patient | Total | E-CTCs | | H-CTCs | | M-CTCs | |
| --- | --- | --- | --- | --- | --- | --- | --- |
| No. | CTCs | GM^-^ | GM^+^ | GM^-^ | GM^+^ | GM^-^ | GM^+^ |
| *Non-metastatic group* | | | | | | | |
| N1 | 0 | 0 | 0 | 0 | 0 | 0 | 0 |
| N2 | 0 | 0 | 0 | 0 | 0 | 0 | 0 |
| N3 | 0 | 0 | 0 | 0 | 0 | 0 | 0 |
| N4 | 0 | 0 | 0 | 0 | 0 | 0 | 0 |
| N5 | 1 | 0 | 0 | 0 | 1 | 0 | 0 |
| N6 | 1 | 0 | 0 | 1 | 0 | 0 | 0 |
| N7 | 2 | 0 | 0 | 0 | 2 | 0 | 0 |
| N8 | 2 | 1 | 0 | 0 | 0 | 1 | 0 |
| N9 | 3 | 0 | 0 | 1 | 1 | 1 | 0 |
| N10 | 3 | 0 | 0 | 0 | 2 | 1 | 0 |
| N11 | 6 | 0 | 1 | 1 | 2 | 1 | 1 |
| N12 | 7 | 0 | 0 | 5 | 2 | 0 | 0 |
| N13 | 8 | 6 | 0 | 2 | 0 | 0 | 0 |
| N14 | 6 | 0 | 0 | 4 | 1 | 1 | 0 |
| N15 | 5 | 0 | 0 | 0 | 2 | 2 | 1 |
| N16 | 3 | 3 | 0 | 0 | 0 | 0 | 0 |
| N17 | 3 | 0 | 1 | 0 | 0 | 1 | 1 |
| N18 | 2 | 1 | 0 | 0 | 1 | 0 | 0 |
| N19 | 2 | 0 | 0 | 2 | 0 | 0 | 0 |
| N20 | 1 | 0 | 0 | 0 | 1 | 0 | 0 |
| N21 | 1 | 1 | 0 | 0 | 0 | 0 | 0 |
| N22 | 0 | 0 | 0 | 0 | 0 | 0 | 0 |
| N23 | 0 | 0 | 0 | 0 | 0 | 0 | 0 |
| N24 | 0 | 0 | 0 | 0 | 0 | 0 | 0 |
| N25 | 0 | 0 | 0 | 0 | 0 | 0 | 0 |
| *Metastatic group* | | | | | | | |
| Y1 | 0 | 0 | 0 | 0 | 0 | 0 | 0 |
| Y2 | 1 | 0 | 0 | 1 | 0 | 0 | 0 |
| Y3 | 2 | 0 | 0 | 1 | 1 | 0 | 0 |
| Y4 | 2 | 0 | 1 | 1 | 0 | 0 | 0 |
| Y5 | 3 | 1 | 0 | 1 | 0 | 1 | 0 |
| Y6 | 5 | 0 | 0 | 1 | 0 | 4 | 0 |
| Y7 | 5 | 0 | 1 | 1 | 0 | 2 | 1 |
| Y8 | 6 | 0 | 0 | 1 | 4 | 0 | 1 |
| Y9 | 7 | 0 | 0 | 2 | 2 | 2 | 1 |
| Y10 | 7 | 0 | 0 | 3 | 4 | 0 | 0 |
| Y11 | 8 | 1 | 1 | 2 | 3 | 1 | 0 |
| Y12 | 11 | 1 | 1 | 1 | 3 | 3 | 2 |
| Y13 | 16 | 2 | 1 | 4 | 9 | 0 | 0 |
| Y14 | 25 | 2 | 0 | 8 | 11 | 2 | 2 |
| Y15 | 33 | 16 | 0 | 14 | 2 | 1 | 0 |
| Y16 | 24 | 0 | 3 | 5 | 13 | 0 | 3 |
| Y17 | 19 | 1 | 5 | 0 | 8 | 3 | 2 |
| Y18 | 9 | 3 | 0 | 2 | 2 | 0 | 2 |
| Y19 | 8 | 8 | 0 | 0 | 0 | 0 | 0 |
| Y20 | 8 | 2 | 1 | 2 | 3 | 0 | 0 |
| Y21 | 7 | 2 | 0 | 1 | 4 | 0 | 0 |
| Y22 | 5 | 0 | 0 | 0 | 2 | 1 | 2 |
| Y23 | 5 | 1 | 1 | 0 | 1 | 0 | 2 |
| Y24 | 5 | 0 | 0 | 2 | 3 | 0 | 0 |
| Y25 | 4 | 1 | 0 | 1 | 1 | 0 | 1 |
| Y26 | 2 | 1 | 0 | 0 | 1 | 0 | 0 |
| Y27 | 2 | 1 | 0 | 0 | 1 | 0 | 0 |
| Y28 | 2 | 1 | 0 | 0 | 1 | 0 | 0 |
| Y29 | 1 | 0 | 1 | 0 | 0 | 0 | 0 |
